# Therapeutic Landscape of Malignant Pleural Mesothelioma: Collateral Vulnerabilities and Evolutionary Dependencies in the Spotlight

**DOI:** 10.3389/fonc.2020.579464

**Published:** 2020-09-23

**Authors:** Duo Xu, Haitang Yang, Ralph A. Schmid, Ren-Wang Peng

**Affiliations:** ^1^Division of General Thoracic Surgery, Inselspital, Bern University Hospital, University of Bern, Bern, Switzerland; ^2^Department for BioMedical Research (DBMR), Inselspital, Bern University Hospital, University of Bern, Bern, Switzerland

**Keywords:** malignant pleural mesothelioma, tumor suppressors, collateral and evolutionary vulnerabilities, targeted therapy, CRISPR/Cas9

## Abstract

Malignant pleural mesothelioma (MPM) is the epitome of a recalcitrant cancer driven by pharmacologically intractable tumor suppressor proteins. A significant but largely unmet challenge in the field is the translation of genetic information on alterations in tumor suppressor genes (TSGs) into effective cancer-specific therapies. The notion that abnormal tumor genome subverts physiological cellular processes, which creates collateral vulnerabilities contextually related to specific genetic alterations, offers a promising strategy to target TSG-driven MPM. Moreover, emerging evidence has increasingly appreciated the therapeutic potential of genetic and pharmacological dependencies acquired en route to cancer development and drug resistance. Here, we review the most recent progress on vulnerabilities co-selected by functional loss of major TSGs and dependencies evolving out of cancer development and resistance to cisplatin based chemotherapy, the only first-line regimen approved by the US Food and Drug Administration (FDA). Finally, we highlight CRISPR-based functional genomics that has emerged as a powerful platform for cancer drug discovery in MPM. The repertoire of MPM-specific “Achilles heel” rises on the horizon, which holds the promise to elucidate therapeutic landscape and may promote precision oncology for MPM.

Malignant pleural mesothelioma (MPM) is a rare but highly aggressive cancer etiologically associated with asbestos exposure and inherently resistant to treatment options (1). Although asbestos is banned in most industrialized countries, MPM incidence and mortality still increase globally owing to long latency of the disease (up to 50 years) and continued use of asbestos in developing countries (2).

For patients with advanced, unresectable MPM, a chemotherapy regimen that combines cisplatin and pemetrexed has for long been the only FDA (U.S. Food and Drug Administration) – approved first-line treatment, which, disappointingly, elicits only modest efficacy due to prevalence of drug resistance and no validated treatment beyond front-line therapy has emerged. However, a recent phase 3 trial has showed that overall survival of MPM patients can be further improved by cisplatin/pemetrexed plus bevacizumab, an antibody against vascular endothelial growth factor (VEGF) (3).

Comprehensive genomic studies have revealed frequent deletions or loss-of-function mutations of tumor suppressor genes (TSGs) in MPM, most often cyclin-dependent kinase inhibitor 2A (*CDKN2A*), BRCA1 associated protein-1 (*BAP1*) and neurofibromatosis type 2 (*NF2*) (4, 5) (Figure 1A), for which direct targeting has proven difficult, contrasted to oncogene-driven malignancies that benefit from a vast majority of molecular targeted anti-cancer drugs. However, aberrant cancer genome rewires biochemical networks, leading to synthetic lethal or collateral vulnerabilities that are contextually linked with specific genetic alterations, providing alternative approaches for targeting TSG-driven MPM (Figure 2). Moreover, cancer-specific dependencies that evolve out of tumor deveoplment and drug resistance offers additional dimensions to expand therapeutic arsenal for MPM. Here, we review the current knowledge about collateral vulnerabilities and evolutionary dependencies in MPM, with an emphasis on the clinical implications for better treatment of the disease (Figure 3 and Table 1).

**FIGURE 1:**
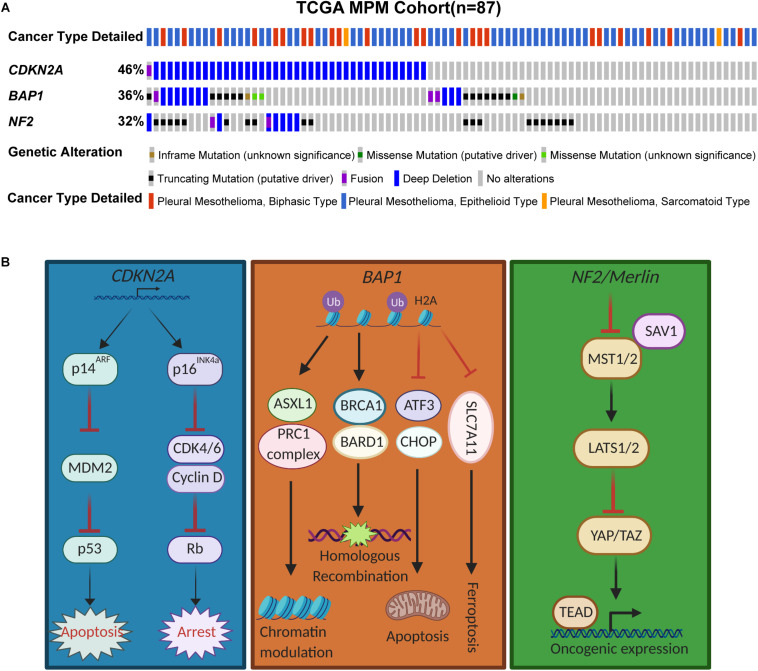
Genomic landscape of *CDKN2A*, *BAP1*, and *NF2* in MPM. **(A)** Somatic mutations of *CDKN2A*, *BAP1*, and *NF2* in The Cancer Genome Atlas (TCGA) samples of MPM patients (*n* = 87). Data were downloaded from cBioPortal. **(B)** Schemtatic illustrating the pathways mediated by the tumor suppressor proteins.

**FIGURE 2:**
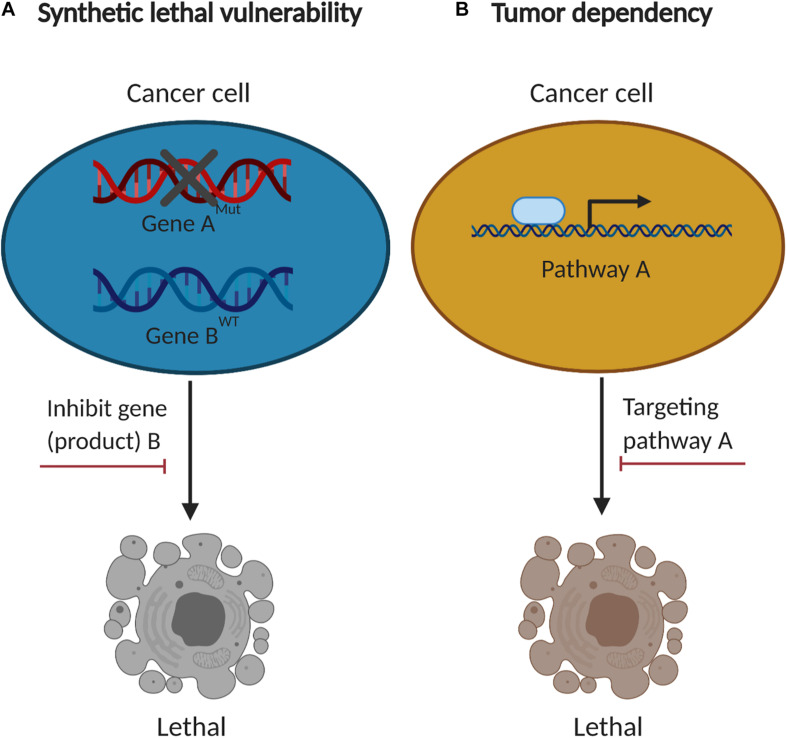
The concept of synthetic lethal vulnerabilities and cancer-specific dependencies. **(A)** Cancer cells with functional loss of a tumor suppressor (gene “A”) are contextually sensitive to inhibition of another gene product (gene “B”). **(B)** Cancer-specific dependencies co-opted by tumorigenesis are compelling therapeutic targets.

**FIGURE 3:**
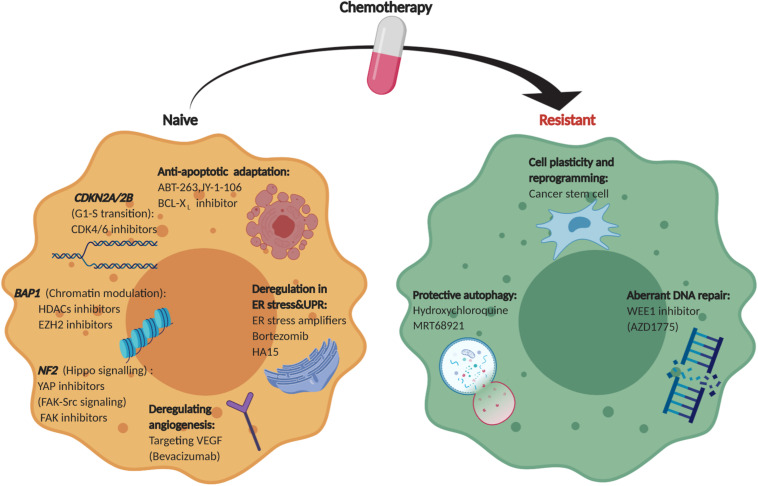
Therapeutic landscape of MPM. An overview of collateral vulnerabilities and evolutionary dependencies identified in therapy-naïve and chemo-resistant MPM.

**TABLE 1:** Clinical trials investigating collateral vulnerabilities and evolutionary dependencies in MPM.

TSG/dependency	Target	Phase	No. patients	Results/trial status	Endpoint (experimental vs. control arms)	Biomarkers (Genetic or molecular)
***CDKN2A/2B***						
Ribociclib (NCT02187783)	CDK4/6	Phase 2	106	Completed (January, 2018)	ORR	CDK4/6, CCND1/3,CDKN2A
Abemaciclib (NCT03654833)	CDK4/6	Phase 2	120	Recruiting	DCR	p16INK4A
***BAP1***						
Vorinostat (27) (NCT00128102)	HDAC	Phase 3	661	Negative (November, 2011)	OS (30.7 vs. 27.1)	None
Tazemetostat (129) (NCT01897571)	EZH2	Phase 1	58	Positive (September, 2016)	Safety	None
Tazemetostat (NCT02860286)	EZH2	Phase 2	67	Completed (May, 2019)	Safety/DCR	BAP1
Niraparib (NCT03207347)	PARP1/2	Phase 2	57	Recruiting	ORR	BAP1/DDR
***NF2***						
Defactinib (49) (NCT01870609)	FAK	Phase 2	372	Negative (January, 2016)	PFS (4.1 vs.4.0) OS (12.7 vs.13.6)	None
**RTKs**						
Erlotinib (NCT00039182)	EGFR	Phase 2	63	Negative (June, 2007)	OS	None
LY3023414 (NCT01655225)	PI3K/mTOR	Phase 1	156	Active Not Recruiting	Safety	None
Everolimus (NCT01655225)	mTOR	Phase 2	59	Negative (September, 2011)	4-month PFS	None
Tivantinib (NCT02049060)	Met	Phase1/2	31	Active Not Recruiting	Safety	None
Bemcentinib (NCT03654833)	AXL	Phase 2	120	Recruiting	DCR	None
**Angiogenesis**						
Thalidomide (76)	VEGF	Phase 3	222	Negative (December, 2009)	Progression (3.6 vs.3.5)	None
Bevacizumab (3) (NCT00651456)	VEGF	Phase 3	448	Positive (September, 2016)	OS (18.8 vs.16.1)	None
**Deregulated UPR**						
Bortezomib (130) (NCT00513877)	Proteasome	Phase 2	33	Negative (December, 2009)	CR/PR	None
Ganetespib (131) (NCT01590160)	HSP90	Phase1/2	27	Completed (November, 2019)	Safety PFS	None

*Abbreviations: CR/PR, complete response/partial response; DCR, disease control rate; ORR, overall response rate; OS, overall survival; PFS, progression-free survival.*

## Collateral Vulnerabilities Caused by Inactivation of Tumor Suppressors

### CDKN2A

The 9p21 locus harboring *CDKN2A* is frequently inactivated in mesothelioma (6). *Cdkn2a* inactivation is a key driver of MPM in a conditional mouse model (7), and *CDKN2A* loss is associated with shorter survival in patients with MPM (8). *CDKN2A* encodes two important tumor suppressor proteins via alternative open reading frames, p16^*Ink4a*^ and p14^*Arf*^ that govern the activity of the retinoblastoma (pRb) and p53 pathways, respectively (9). While the absence of p16^*Ink4a*^, an inhibitor of cyclin-dependent kinases 4/6 (CDK4/6)-mediated phosphorylation of pRb, abrogates the G1/S cell-cycle arrest and promotes aberrant proliferation, functional loss of p14^*Arf*^, a central negative regulator of mouse double minute 2 homolog (MDM2), suppresses apoptosis by escape from p53-mediated anti-tumor surveillance (Figure 1B).

Recent studies showed that *CDKN2A* deficiency render MPM cells particularly vulnerable to CDK4/6-targeted therapies, and that targeting PI3K further enhances the efficacy by precluding resistance to CDK4/6 inhibition (10). CDK4/6 inhibitors, e.g., palbociclib, ribociclib, and abemaciclib, are FDA-approved drugs that provide readily translatable therapeutics for MPM. Clinical trial for efficacy of Ribociclib in CDK4/6 pathway activated hematologic malignancies and solid tumors including MPM has been completed (NCT02187783). Abemaciclib as monotherapy is being investigated in MPM bearing p16^*Ink4a*^ deficiency (Clinicaltrials.gov no. NCT03654833).

Strikingly, in around 30% MPM cases *CDKN2A* alterations co-occur with biallelic deletion in type I interferon (IFN-I; mainly IFN-α and IFN-β) locus. The IFN-I pathway plays a key antiviral role, suggesting that *CDKN2A*-deficient MPM might particularly benefit from oncolytic viral therapy (11), a novel anti-cancer strategy that exploits oncolytic viruses, which preferentially replicate in cancer but not in normal cells (12).

### BAP1

*BAP1*, originally identified as an associated protein of the breast cancer susceptibility gene product BRCA1, is a nuclear deubiquitinase with ubiquitin carboxy-terminal hydrolase activity and contributes to the inhibition of E3 ligase function of BRCA1/BRAD during DNA damage response (13) (Figure 1B). Germline mutations in *BAP1* causes a predisposition syndrome with increased risk to renal cell carcinoma, MPM and uveal melanoma (14). *BAP1* is also frequently inactivated by somatic mutations and loss of the 3p21.1 locus in MPM (15) (Figure 1A). Other mechanisms leading to inactivation of *BAP1* includes chromosomal rearrangements, gene fusion and splice alterations (4).

As a component of the polycomb repressive deubiquitinase (PR-DUB) complex, *BAP1* deletion causes defects in homologous recombination (HR), contributed by failure to deubiquitinate histone H2A on chromatin, which eventually leads to accumulation of DNA mutations and chromosomal aberrations (16). BAP1 has also been shown to regulate cell cycle through interactions with transcription regulators such as host cell factor-1 (HCF-1) and E2F transcription factor 1 (E2F1) (17). As *BAP1* loss causes deficient HR, *BAP1*-mutant tumors were reported to be particularly addicted to alternative DNA repair pathways, e.g., PARP1-mediated ones (16, 18). However, the sensitivity of mesothelioma cells to PARP inhibitors may be independent of the *BAP1* status (19, 20), warranting further studies to investigate the BAP1/PARP intersection. Interestingly, TNF-related apoptosis-inducing ligand (TRAIL) has been shown highly effective for *BAP1*-deficient MPM, likely via modulating the PR-DUB activity (21).

The heterozygous germline *BAP1* mutations were reported to increase aerobic glycolysis, also known as the “Warburg effect,” due to impaired mitochondrial respiration (22), suggesting a role for BAP1 in metabolism. Indeed, recent evidence has indicated that loss of *BAP1* promotes cellular adaptability to metabolic stress by impairing ER stress gene regulatory network (e.g., *ATF3* and *CHOP*) and ferroptosis via modulating SLC7AL11 (23, 24). Informed by these findings, signaling pathways that regulate metabolic stress might be promising targetable vulnerabilities in *BAP1*-deficient MPM.

BAP1 has also been implicated in chromatin modulation by interacting with ASXL1 and polycomb repressive complex 1 (PRC1) (25), and further preclinical evidence revealed that *BAP1* loss renders MPM sensitivity to histone deacetylases (HDACs) inhibitors (26). However, a large phase 3 trial of MPM (*n* > 600) with Vorinostat, an FDA-approved HDAC inhibitor, showed disappointing results (27). Importantly, recent studies indicate that *BAP1* loss in MPM prioritizes the enhancer of zeste homolog 2 (EZH2)-targeted therapy (28), suggesting that aberrant expression of EZH2, known to lead to uncontrolled cell proliferation and tumorigenesis (29), might be an inherent feature enabled by the suppression of BAP1. Strikingly, a phase II study showed that tazemetostat, an oral EZH2 inhibitor, demonstrated promising clinical benefit in patients with malignant mesothelioma (30). Notably, it has been reported that sensitivity of uveal melanoma to EZH2 inhibitors is independent of *BAP1* mutational status (31), suggesting a lineage-specific effect of BAP1 on the activity of EZH2i (EZH2 inhibitors).

Surprisingly, several studies have revealed that *BAP1* mutaions in MPM are associated with favorable prognosis in patients (32, 33). One possible explanation is that BAP1 loss is accompanined with impaired DNA repair capacity, which enhances sensitivity to chemotherapy and thus may improve patients’ outcome. In addition, *BAP1* deletion is linked with fewer chromosome arm gain and loss, as well as less somatic copy number alterations (SCNAs) (4, 5). Finally, *BAP1*-inactivated tumors might have a stronger activity of cytotoxic T cells due to increased interferon regulator factor 8 (IRF8) (5, 34).

### NF2

*NF2* encodes Merlin (moesin-ezrin-radixin-like protein) that mediates tumor suppression and contact-dependent inhibition through the Hippo pathway (35) (Figure 1B).

The Hippo pathway is evolutionally conserved that regulates organ size, tissue homeostasis and apoptosis, best characterized as a downstream transducer of Merlin/NF2 signaling (36). Merlin exerts tumor-suppressor function by translocating to the nucleus, where it promotes the degradation of two paralogous transcriptional co-activators, Yes-associated protein (YAP) and WWTR1 (WW domain containing transcription regulator 1)/transcriptional coactivator with PDZ-binding motif (TAZ) via the E3 ubiquitin ligase CRL4^*DCAF1*^ (37). Merlin also activates serine/threonine kinase macrophage stimulating 1/2 (MST1/2), causing large tumor suppressor kinase 1/2 (LATS1/2)-dependent phosphorylation and degradation of YAP/TAZ (38). In addition to *NF2* mutations (Figure 1A), gene fusion and chromosomal rearrangements also result in nonfunctional NF2 and therefore inactivates Hippo pathway in MPM (4). In addition, other components of the Hippo pathway, such as *LATS2*, are also frequently inactivated in MPM (4), which inactivates the Hippo pathway as well. *LATS2* alterations (mutations, copy loss) occur in about 11% of MPM patients (5), and *LATS2*-deficient MPM might have similar vulnerabilities as those where the Hippo pathway is inactivated via other mechanisms. In particular, MPM with co-occuring mutations in *LATS2* and *NF2* has been reported to rely on an altered mTOR/PI3K/AKT signaling, and therefore highly sensitive to PF-04691502, a potent and selective oral inhibitor of PI3K and mTOR kinases (39). Finally, gene fusions involving *LIFR*, encoding a metastasis suppressor, was identified as an additional mechanism that inactivates Hippo pathway in MPM (4). The disrupted Hippo pathway constitutively activates oncogenic YAP/TAZ, which in turn transcriptionally activates cancer-promoting genes through interaction with TEA/ATTS domain (TEAD) transcription factors (40). Supporting this notion, verteporfin, an inhibitor against the YAP/TEAD interaction, exerts strong anti-MPM effects, suggesting that the YAP/TEAD axis might be a collateral vulnerability in *NF2*-deficient MPM (41). However, some other studies have shown that response to verteporfin can be independent on YAP activity (42, 43), indicating that the exact correlation between YAP activity and drug response remains to be determined. Further, inhibition of YAP/TAZ was reported to compensatorially activate the MAPK pathway and, as a consequence, co-targeting YAP/TAZ and MEK, a key constituent of the MAPK signaling cascade, synergizes in suppressing the growth of *NF2*-deficient tumors (44), providing a rational combination strategy for *NF2*-mutant MPM.

Finally, NF2/Merlin inactivation has been associated with upregulation of the focal adhesion kinase (FAK) activity in mesothelioma (45). FAK is a cytoplasmic tyrosine kinase that integrates signals from integrins and growth factor receptors to multiple cellular processes, ranging from cell proliferation and migration to renewal of cancer stem cells and resistance to chemotherapy (46). Merlin levels are predictive of sensitivity of MPM cells to the FAK inhibitor VS-4718 (47). Notably, the sensitivity to FAKi has also been shown independent of NF2/Merlin inactivation, and E-cadherin has been reported to be a predictive biomarker for FAK-targeted therapy (43, 48). Intriguingly, although a phase 1 study with FAK inhibitors showed longer median progression-free survival (PFS) in patients with NF2-negative MPM than those with NF2-positive tumors (49), a large phase II COMMAND trial demonstrated that neither PFS nor OS (overall survival) was improved by FAK inhibitors in patients with NF2-low MPM and prior treatment with chemotherapy (50). Together, these results indicate that further investigations are required to establish the link between NF2 mutational status and response to FAK inhibition.

### Other Genetic Alterations in MPM

In addition to the TSGs (*CDKN2A*, *BAP1*, and *NF2*) described above, several other genes, e.g., *TP53* (tumor protein p53), *LATS2*, *SETD2* (SET domain containing 2) and oncogenic changes in the *TERT* (telomerase reverse transcriptase) promoter are also altered by at non-negligible frequencies in MPM.

*TP53* (encoding p53) is mutated in 6–16% MPM cases (51). Moreover, *CDKN2A* loss that depletes p14^*Arf*^ and in turn releases MDM2 (mouse double minute 2 homolog), a negative regulator of p53, also inactivates p53, a key player in G1/S cell cycle regulation and apoptosis. We and others have shown that inactivation of *CDKN2A/2B* and *TP53* renders MPM cell particularly sensitive to G2 checkpoint inhibition, e.g., CBP501 (a peptide with G2 checkpoint-abrogating activity) and AZD1775 (selective inhibitor of the G2 checkpoint kinase WEE1) (52, 53).

Genetic alterations (mutation, gene fusion and splice alteration) in *SETD2*, an epigenetic tumor suppressor involved in histone methylation, are detected in more than 8% of MPM cases (4). *SETD2*-deficient MPM may respond favorably to inhibitors of histone methyltransferase EZH2 (54). In addition, synthetic lethality between *SETD2* deficiency and CDK7 inhibitor THZ1 has been recently reported in kidney cancer (55), although a similar efect on mesothelioma remains to be determined.

*TERT* promoter mutations, the first recurrent oncogenic muation identified in MPM, are frequent in MPM with sarcomatoid subtype and significantly associated with worse clinical outcome (56, 57). As expected, MPM cells carrying *TERT* promoter mutations show increased sensitivity to telomerase inhibition than those with wide-type *TERT*, indicating that targeting telomerase activity might be a promising treatment strategy for this MPM subset (57).

## Genetic and Pharmacological Dependencies Evolve En Route to Cancer Development

In contrast with normal cells, tumor cells acquire unchecked cell growth en route to cancer development, a result of persistent activation of oncogenic pathways that concomitantly increases the level of cellular stresses (58). To deal with the stressful stimuli, cancer cells require the activity of a plethora of genes and cellular processes that become necessary to cancer cell survival. This phenomenon is referred to as non-oncogene addiction or cancer dependency (59), which represents the “Achilles’ heel” of a specific type of cancers that can be therapeutically exploited without impairing normal cells. Recent studies have identified several genetic and pharmacological dependencies in MPM, covering various pathways involved in receptor kinase signaling, DNA damage repair and proteotoxic stress.

### Receptor Tyrosine Kinases (RTKs)

Self-sufficiency in proliferative signaling, often through aberrant activation of receptor tyrosine kinases (RTKs), is a hallmark of cancer including MPM. Epidermal growth factor receptor (EGFR) is not mutated but overexpressed in MPM, leading to deregulation of EGFR signaling and aberrant activation of downstream pathways such as RAS/RAF/MAPK and PI3K/AKT/mTOR, which in turn promotes cell proliferation, tumor invasiveness and angiogenesis (60). Despite the high expression of EGFR, no clinical benefit was observed in MPM patients treated with erlotinib, an EGFR-specific inhibitor (61). Trametinib, a specific MEK inhibitor, exhibited anti-tumor effects in MPM, and displayed strong synergestic effects when combined with the FAK inhibitor GSK2256098 (62). Emerging evidence has also shown that multipoint targeting of PI3K/AKT/mTOR pathway is a promising strategy for therapeutic intervention of MPM (63). Although mTOR inhibition with specific inhibitors suppressed mesothelioma cell growth in pre-clinical models (64, 65), only limited clinical activity was observed in patients with advanced MPM (66). Additionally, preclinical studies identified MET and AXL as putative therapeutic targets in MPM (67, 68), and clinical trials testing Met-specific TKI (tivantinib; NCT02049060) and AXL-specific TKI (bemcentinib; NCT03654833) are ongoing.

Increasing evidence has indicated that other RTKs, including fibroblast growth factor receptor (FGFR) (69), insulin-like growth factor 1 receptor (IGF1R) (70), platelet-derived growth factor receptor (PDGFR) (71) and vascular endothelial growth factor receptor (VEGFR) are also aberrantly activated in MPM (72). In particular, preclinical studies have revealed that imatinib, a multi-target inhibitor of v-ABL, c-KIT and PDGFRβ, enhanced therapeutic effects of chemotherapeutic drugs (gemcitabine, pemetrexed) via AKT inactivation in malignant mesothelioma, which has encouraged the initiation of clinical trials (73, 74).

Given the crucial role of VEGF signaling in tumorigenesis, several anti-angiogenic drugs, including bevacizumab, thalidomide and nintedanib, have been investigated in MPM either alone or in combination with cisplatin plus pemetrexed over the past decade (75). To date, three randomized phase 3 studies that assess anti-angiogenic inhibitors in patients with MPM have been performed. In the 2013 study, no clinical benefit was observed for the addition of thalidomide to first-line chemotherapy (76), while the large randomized Mesothelioma Avastin Cisplatin Pemetrexed Study (MAPS) in 2016 showed that the triple combination of bevacizumab, cisplatin and pemetrexed significantly improved the median overall survival (OS) in MPM (3). More recently, another randomized phase 3 trial demonstrated no benefits of nintedanib addition compared to standard chemotherapy alone (77). The varied results highlight the need of further stratification for anti-angiogenic therapeutics in MPM.

### Endoplasmic Reticulum (ER) Stress and Unfolded Protein Response (UPR)

The endoplasmic reticulum (ER) is the principal organelle monitoring proteostasis. Physiological and pathologic stimuli e.g., nutrient deprivation, aberrant glycosylation, oxidative stress and DNA damage, can disturb the ER environment, eliciting ER stress and unfolded protein response (UPR) (78). The UPR is mediated by three effector arms: double-stranded RNA-activated protein kinase (PKR)-like ER kinase (PERK), inositol-requiring enzyme 1α (IRE1α), and activating transcription factor 6 (ATF6) (79). At the steady state, the chaperone protein glucose-regulated protein 78 (GRP78, also known as BiP) binds and represses PERK, IRE1α and ATF6. When threatened by increased protein-folding demand (ER stress), BiP is released, which activates PERK, IRE1α, and ATF6, and in turn, their downstream effectors to alleviate proteotoxic stress placed on the ER and restore proteostasis in the ER (80).

Tumorigenesis entails aberrant proliferation, which is often confronted by limited oxygen supply and malfunctional vascularization, leading to increased demand for protein folding, assembly and transport (81). As such, the ER stress response or UPR is a cytoprotective mechanism that promotes survival and adaptation to adversary environment, which might render tumor cells particularly vulnerable to agents that further disturb ER stress. Indeed, therapeutic targeting of ER stress/UPR pathway has emerged as a promising strategy of cancer treatment (82). There is a positive correlation between expression of the ER stress-responsive phosphatase growth arrest and DNA damage 34 (GADD34) and differentiation status of mesothelial cells (83), suggesting that modulating ER stress might be effective for MPM. Consistently, we have recently showed that deregulated ER stress/UPR is a characteristic feature of MPM, and that therapeutic modulation of the signaling impairs the growth of MPM cells and overcomes resistance to standard chemotherapy (84, 85).

### Anti-apoptotic Adaptation

Escape from apoptosis, a critical barrier of tumor development, is a prominent hallmark of cancer, including MPM. Apart from the loss of *TP53* tumor suppressor functions and increased expression of survival signals, overexpression of pro-survival B-cell lymphoma 2 (BCL-2) family proteins (BCL-2, BCL-X_*L*_, MCL-1, BCL-W, BCL-B, BFL-1) that dampen apoptosis by sequestering pro-apoptotic activators (BAX, BIM, PUMA) is another pivotal strategy to circumvent apoptosis (86). In this scenario, cancer cells are thought to be “primed” for apoptosis, as they accumulate the pro-apoptotic activators (87). This trait can be exploited for cancer treatment by blocking specific or multiple pro-survival proteins with B-cell lymphoma 2 (Bcl-2) homology 3 (BH3)-mimetic therapy that overwhelms the anti-apoptotic defenses (88). In MPM, pro-survival or apoptosis suppression has been reported to be promoted by defects in core-apoptosis signaling (89), and the BH3 mimetic ABT-737 (targeting BCL-2/BCL-X_*L*_/BCL-W) (90) and a pan-BCL-2 inhibitor (JY-1-106) are active against MPM cells (91, 92). More recently, BCL-X_*L*_ has been identified as a key anti-apoptotic mediator in MPM cells, further highlighting the promise of therapeutic strategies that modulate apoptotic threshold in MPM (93).

### Tumor Microenvironment (TME)

A key feature of tumor microenvironment is hypoxia, which promotes acquisition of aggressive phenotypes. Emerging evidence has suggested that hypoxia-inducible factors (HIF-1α, -2α) play central roles in regulating hypoxic responses in MPM (94). HIF-1α/2α are transcription factors induced by hypoxic conditions, which in turn alter the expression of various target genes, such as those encoding the stem-like factor OCT4, anti-apoptotic BCL-2, glucose transporter 1 (GLUT1), VEGF, E-Cadherin and Vimentin, leading to the change of diverse biological functions, e.g., angiogenesis, anti-apoptosis, cell motility and metabolism (95, 96). Moreover, hypoxia is associated with increased genome instability by downregulating several DNA repair genes such as *MLH2*, *MSH2*, and *RAD51* (97). Thus, targeting tumor hypoxia might be a promising strategy for treating MPM.

### A Roadmap to Cancer Dependencies

The Cancer Dependency Map Project (DepMap)^1^ is dedicated to systematic identification of cancer-specific vulnerabilities for targeted therapy and further stratification based on genomic diversity and molecular characteristics for precision oncology (98). DepMap provides a range of information for the genetic landscape, expression profile, genetic essentiality and drug sensitivity across a broad spectrum of human cancers including MPM by incorporating The Cancer Cell Line Encyclopedia (CCLE), Project Achilles and PRISM, which may facilitate the prioritization of therapeutic targets for the development of precision cancer medicines.

## Evolutionary Vulnerabilities Co-Opted by Chemotherapy Resistance

Despite decades of enormous efforts, cisplatin plus pemetrexed chemotherapy remains one of the few treatment options that achieve survival benefit in MPM. However, clinical evidence indicates that this combination therapy rarely achieves complete/durable clinical response in MPM patients due to drug resistance, intrinsic and/or acquired after initial treatment. Therefore, identification of therapeutic vulnerabilities to target chemoresistant MPM represents a significant yet unmet clinical challenge.

### Cancer Cell Plasticity and Cancer Stem Cells

Cancer cells can shift at different cell states, most prominently the transition from epithelial to mesenchymal (EMT) or vice versa (MET). The epithelial state is a differentiated cell state while the mesenchymal more undifferentiated and reminiscent of cancer stem-like cells (CSCs), so coined as they recapitulate normal stem cells characterized by the capacity of self-renewal and differentiation. Cancer cell plasticity is an important process that generates CSCs and drives therapy resistance (99), partly due to relative dormancy of slowing cell-cycle kinetics, efficient DNA repair capacity and expression of multidrug-resistance transporters and resistance to apoptosis of the cells (100).

Putative CSCs identified in MPM express high levels of CD24, ABCG2, ABCB5 and OCT4 and confer drug resistance (101). We have showed that MPM cells populated as spheroids are highly resistant to standard chemotherapy (84). Mesothelioma stem cells (MSCs) might be responsible for tumor repopulation after chemoradiation in murine mesothelioma (102), and cisplatin-resistant, CSC-like subpopulations could be enriched by aldehyde dehydrogenase (ALDH)^*high*^ and CD44^+^ in mesothelioma cell lines (103). Despite the progress, the molecular mechanisms underlying cancer cell plasticity and the malignancy of CSCs remain incompletely understood.

### Aberrant DNA Repair

The DNA damage response (DDR) synchronizes DNA repair and checkpoint signaling activation to arrest cell cycle progression (104). Compelling evidence suggests that DDR protects against genomic instability and affects responses to genotoxic agents (105), although loss of some elements involved in DNA repair pathways is predisposed to malignancy. Conversely, upregulated DDR signaling confers resistance to DNA- damaging chemotherapy and inhibitors targeting the altered pathways have the potential to revert resistance and augment the efficacy of conventional chemotherapy (106). MPM patients with germline mutations in *BAP1* or other DNA repair genes (*CHEK2*, *PALB2*, *BRCA2*, and *MLH1*) show increased sensitivity to cytotoxic chemotherapy due to impaired DDR (107). Moreover, the Fanconi anemia (FA)/BRCA2 pathway involved in the homologous recombination DNA repair has been shown to play a key role in MPM chemoresistance (108), and a potential link of deregulated G2/M checkpoint pathway with tumor progression and sensitivity to chemotherapeutic agent cisplatin has been proposed (109). Notably, p53 signaling is frequently inactivated in MPM (4, 51), a consequence of *TP53* mutaions (6–16% in MPM) and, more often, of inactivating alterations in *CDKN2A*, which depletes p14^*Arf*^ and promotes proteasome-mediated degradation of p53. Consequently, p53 deficient MPM cells might have greater dependence on G2/M checkpoint to protect the toxicity of chemotherapy, and abrogation of the G2/M checkpoint activity, e.g., WEE1 inhibition, sensitizes MPM cells to chemotherapy (53, 110).

### Autophagy

Macroautophagy (hereafter autophagy) is an evolutionally conserved catabolic process, whereby long-lived proteins and damaged organelles are sequestered in a double-membraned vesicle (autophagosome) and delivered to the lysosomes for degradation and recycled to fuel cellular growth (111). Autophagy is regulated by a variety of autophagy-associated genes (ATGs), with its contribution to cancer being controversial, as autophagy can be either pro-survival (oncogenic) or tumor suppressive at different stages of cancer progression (112). It has been reported that MPM cells display a generally high basal level of autophagy, which is critical for tumor growth (113). Albeit autophagic cell death (also known as type II programmed cell death) is potentially exploitable as an anticancer therapy, the vast majority of studies have demonstrated that autophagy is a protective mechanism linked with increased resistance to chemo and targeted therapy (114, 115). Supporting this notion, autophagy inhibition was shown to effectively improve chemosensitivity in mesothelioma (116).

## Systematic Approaches for Cancer Drug Discovery in MPM

### RNA Interference (RNAi) Screening

RNAi confers transient or stable gene silencing by small interfering RNAs (siRNA) or short hairpin RNAs (shRNA). Genome-wide screens with pooled shRNA libraries are widely pursued to identify cancer drivers and context-dependent events such as synthetic lethal interactions and collateral vulnerabilities (117). Experimentally, cancer cells infected with shRNA vectors that are specifically identified by molecular barcodes are monitored for growth and subsequently subjected to genomic DNA isolation, polymerase chain reaction (PCR) amplification and quantification of the molecular barcodes. Comparison of barcode abundance between experimental and reference samples, genes required for cancer cell proliferation can be identified. Several groups have performed shRNA screes in MPM (118, 119), which however, surveyed relatively few cell lines and none represented the diversity of MPM. Although RNAi is ideal to evaluate the temporary gene disruption (e.g., to mimic the effect of cancer drugs), the utility of RNAi is limited by incomplete silencing, high off-target effects and stimulated immune response.

### CRISPR/Cas9 Mediated Genome Editing

**FIGURE 4:**
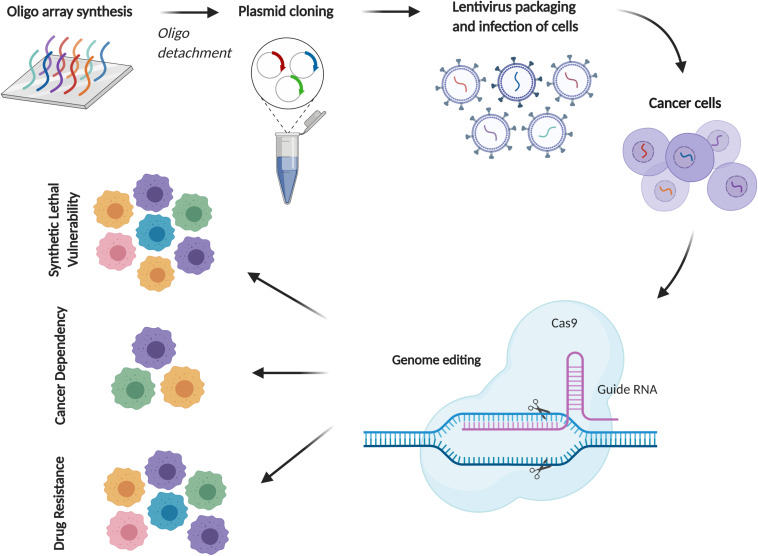
CRISPR/Cas9 screening for cancer drug discovery in MPM. A schematic diagram of CRISPR/Cas9 screening using pooled sgRNA libraries. Depending on the experimental setting, comparison of sgRNA abundance with reference samples can identify essential genes for drug resistance, cell proliferation or context-dependent events (e.g., synthetic lethal vulnerability with specific mutations).

CRISPR/Cas9 (clustered regularly interspaced short palindromic repeats/CRISPR- associated 9) is a gene-editing technology allowing for rapid and accurate assessment of gene functions with fewer off-target effects compared to RNAi, which, due to its scalability, has emerged as an important tool for large-scale screens. Functional genomics using CRISPR have to date focused on identifying genes required by cancer cells for growth or response to a therapy, which provides a novel genetic tool to ascertain gene functions by customizing the single guide RNA (sgRNA) sequence. After transduced by pooled sgRNA library, the recipient cells become genetically heterogeneous, each one with a knockout of a different gene. Following culture and selection, e.g., drug treatment, cells expressing sgRNAs that target the genes essential for proliferation or drug resistance will die, thereby depleting them from residual tumor cells after culture or treatment (Figure 4).

CRISPR screens of genome and customized sgRNA libraries in numerous disease models have identified novel oncogenic drivers and cancer dependencies (120, 121), synthetic lethal interactions with mutant *RAS* and *BRAF* (122, 123) and mechanisms of resistance to anti-cancer drugs (124). Amenability to *in vivo* systems greatly enhances CRISPR applicability in clinically relevant settings (125). CRISPR-based functional genomics in MPM is still at its infancy, we recently screened MPM kinome and delineated that deregulated G2-M checkpoint activity dictates MPM response to chemotherapy (53).

### Further Considerations in Systematic Approaches

MPM displays high heterogeneity, characterized by inter- and intratumor variability at cellular and molecular levels (4, 5, 126, 127). Heterogeneity represents a key mechanism underlying the poor response of MPM patients to current therapeutic interventions and is a challenge in systematic studies aimed at identifying effective treatment and curtailing drug resistance. Notably, cancer cell lines utilized predominantly in current RNAi- and CRISPR-based functional genomic studies poorly recapitulate the condition under which heterogeneous tumors arise and evolve. More instrumental *in vitro* models that more closely capture the heterogeneity of patient samples include cancer stem cells and patient-derived organoids (128). Moreover, *in vivo* models that recapitulate the cellular and genetic complexity of human cancer during tumor evolution and progression amid drug treatment, such as genetically engineered mouse models (GEMMs), syngeneic mouse models, and patient-derived xenografts (PDX), should be considered in systematic approaches (125). Despite in its infancy, future studies taken into account of tumor heterogeneity is likely the only way toward the development of precision medicine for MPM patients.

## Concluding Remarks

Unlike many other solid tumors, MPM is characterized by overwhelming prevalence of loss of function alterations in tumor suppressor genes, for which direct pharmacological targeting proves difficult. However, collateral genotoxic, proteotoxic, and metabolic stresses caused by abnormal tumor genome or anti-cancer drugs can generate context-dependent vulnerabilities and dependencies, which has profound implications for alternate treatment of TSG-driven MPM. Recent studies have identified previously unappreciated vulnerabilities contextually linked with aberrant TSGs and dependencies acquired during cancer development and drug resistance, which provides unprecedented insights into MPM pathobiology and may bring about unprecedented hopes for the development of biomarker-guided precision medicine for the disease (Figure 3). Integrative molecular characterization enabled by large-scale RNA and proteomic profiling studies, partnered by functional genomics, holds importance to completely unfold the therapeutic landscape for MPM.

## Author Contributions

DX conducted literature search, drafted and revised the manuscript, and prepared the figures and table. HY and RS reviewed the manuscript. R-WP designed the study and revised and proofread the manuscript. All authors contributed to the article and approved the submitted version.

## Conflict of Interest

The authors declare that the research was conducted in the absence of any commercial or financial relationships that could be construed as a potential conflict of interest.

## Footnotes

1 https://depmap.org/portal/depmap/

## References

[1] Lin R-T Takahashi K Karjalainen A Hoshuyama T Wilson D Kameda T Ecological association between asbestos-related diseases and historical asbestos consumption: an international analysis. *Lancet.* (2007) 369:844–9. 10.1016/s0140-6736(07)60412-7 17350453

[2] Stayner L Welch LS Lemen R. The worldwide pandemic of asbestos-related diseases. *Annu Rev Public Health.* (2013) 34:205–16. 10.1146/annurev-publhealth-031811-124704 23297667

[3] Zalcman G Mazieres J Margery J Greillier L Audigier-Valette C Moro-Sibilot D Bevacizumab for newly diagnosed pleural mesothelioma in the Mesothelioma Avastin Cisplatin Pemetrexed Study (MAPS): a randomised, controlled, open-label, phase 3 trial. *Lancet.* (2016) 387:1405–14. 10.1016/S0140-6736(15)01238-6 26719230

[4] Bueno R Stawiski EW Goldstein LD Durinck S De Rienzo A Modrusan Z Comprehensive genomic analysis of malignant pleural mesothelioma identifies recurrent mutations, gene fusions and splicing alterations. *Nat Genet.* (2016) 48:407–16. 10.1038/ng.3520 26928227

[5] Hmeljak J Sanchez-Vega F Hoadley KA Shih J Stewart C Heiman D Integrative molecular characterization of malignant pleural mesothelioma. *Cancer Discov.* (2018) 8:1548–65. 10.1158/2159-8290.CD-18-0804 30322867 PMC6310008

[6] Cheng JQ Jhanwar SC Klein WM Bell DW Lee WC Altomare DA p16 alterations and deletion mapping of 9p21-p22 in malignant mesothelioma. *Cancer Res.* (1994) 54:5547–51. 7923195

[7] Jongsma J van Montfort E Vooijs M Zevenhoven J Krimpenfort P van der Valk M A conditional mouse model for malignant mesothelioma. *Cancer Cell.* (2008) 13:261–71. 10.1016/j.ccr.2008.01.030 18328429

[8] Jennings CJ Murer B O’Grady A Hearn LM Harvey BJ Kay EW Differential p16/INK4A cyclin-dependent kinase inhibitor expression correlates with chemotherapy efficacy in a cohort of 88 malignant pleural mesothelioma patients. *Br J Cancer.* (2015) 113:69–75. 10.1038/bjc.2015.187 26057448 PMC4647524

[9] Stott FJ Bates S James MC McConnell BB Starborg M Brookes S The alternative product from the human CDKN2A locus, p14(ARF), participates in a regulatory feedback loop with p53 and MDM2. *EMBO J.* (1998) 17:5001–14. 10.1093/emboj/17.17.5001 9724636 PMC1170828

[10] Bonelli MA Digiacomo G Fumarola C Alfieri R Quaini F Falco A Combined inhibition of CDK4/6 and PI3K/AKT/mTOR pathways induces a synergistic anti-tumor effect in malignant pleural mesothelioma cells. *Neoplasia.* (2017) 19:637–48. 10.1016/j.neo.2017.05.003 28704762 PMC5508477

[11] Delaunay T Achard C Boisgerault N Grard M Petithomme T Chatelain C Frequent homozygous deletions of type I interferon genes in pleural mesothelioma confer sensitivity to oncolytic measles virus. *J Thorac Oncol.* (2020) 15:827–42. 10.1016/j.jtho.2019.12.128 31945495

[12] Pease DF Kratzke RA. Oncolytic viral therapy for mesothelioma. *Front Oncol.* (2017) 7:179. 10.3389/fonc.2017.00179 28884088 PMC5573749

[13] Nishikawa H Wu W Koike A Kojima R Gomi H Fukuda M BRCA1-associated protein 1 interferes with BRCA1/BARD1 RING heterodimer activity. *Cancer Res.* (2009) 69:111–9. 10.1158/0008-5472.CAN-08-3355 19117993

[14] Carbone M Yang H Pass HI Krausz T Testa JR Gaudino G. BAP1 and cancer. *Nat Rev Cancer.* (2013) 13:153–9. 10.1038/nrc3459 23550303 PMC3792854

[15] Bott M Brevet M Taylor BS Shimizu S Ito T Wang L The nuclear deubiquitinase BAP1 is commonly inactivated by somatic mutations and 3p21.1 losses in malignant pleural mesothelioma. *Nat Genet.* (2011) 43:668–72. 10.1038/ng.855 21642991 PMC4643098

[16] Yu H Pak H Hammond-Martel I Ghram M Rodrigue A Daou S Tumor suppressor and deubiquitinase BAP1 promotes DNA double-strand break repair. *Proc Natl Acad Sci USA.* (2014) 111:285–90. 10.1073/pnas.1309085110 24347639 PMC3890818

[17] Eletr ZM Wilkinson KD. An emerging model for BAP1’s role in regulating cell cycle progression. *Cell Biochem Biophys.* (2011) 60:3–11. 10.1007/s12013-011-9184-6 21484256 PMC3128820

[18] Parrotta R Okonska A Ronner M Weder W Stahel R Penengo L A novel BRCA1-associated protein-1 isoform affects response of mesothelioma cells to drugs impairing BRCA1-mediated DNA repair. *J Thorac Oncol.* (2017) 12:1309–19. 10.1016/j.jtho.2017.03.023 28389374

[19] Srinivasan G Sidhu GS Williamson EA Jaiswal AS Najmunnisa N Wilcoxen K Synthetic lethality in malignant pleural mesothelioma with PARP1 inhibition. *Cancer Chemother Pharmacol.* (2017) 80:861–7. 10.1007/s00280-017-3401-y 28756516 PMC5608777

[20] Rathkey D Khanal M Murai J Zhang J Sengupta M Jiang Q Sensitivity of mesothelioma cells to PARP inhibitors is not dependent on BAP1 but is enhanced by temozolomide in cells with high-Schlafen 11and low-MGMT expression. *J Thorac Oncol.* (2020) 15:843–59. 10.1016/j.jtho.2020.01.012 32004714 PMC8437153

[21] Kolluri KK Alifrangis C Kumar N Ishii Y Price S Michaut M Loss of functional BAP1 augments sensitivity to TRAIL in cancer cells. *eLife.* (2018) 7:e30224. 10.7554/eLife.30224 29345617 PMC5773178

[22] Bononi A Yang H Giorgi C Patergnani S Pellegrini L Su M Germline BAP1 mutations induce a Warburg effect. *Cell Death Differ.* (2017) 24:1694–704. 10.1038/cdd.2017.95 28665402 PMC5596430

[23] Dai F Lee H Zhang Y Zhuang L Yao H Xi Y BAP1 inhibits the ER stress gene regulatory network and modulates metabolic stress response. *Proc Natl Acad Sci USA.* (2017) 114:3192–7. 10.1073/pnas.1619588114 28275095 PMC5373337

[24] Zhang Y Shi J Liu X Feng L Gong Z Koppula P BAP1 links metabolic regulation of ferroptosis to tumour suppression. *Nat Cell Biol.* (2018) 20:1181–92. 10.1038/s41556-018-0178-0 30202049 PMC6170713

[25] Campagne A Lee MK Zielinski D Michaud A Le Corre S Dingli F BAP1 complex promotes transcription by opposing PRC1-mediated H2A ubiquitylation. *Nat Commun.* (2019) 10:348. 10.1038/s41467-018-08255-x 30664650 PMC6341105

[26] Sacco JJ Kenyani J Butt Z Carter R Chew HY Cheeseman LP Loss of the deubiquitylase BAP1 alters class I histone deacetylase expression and sensitivity of mesothelioma cells to HDAC inhibitors. *Oncotarget.* (2015) 6:13757–71. 10.18632/oncotarget.3765 25970771 PMC4537048

[27] Krug LM Kindler HL Calvert H Manegold C Tsao AS Fennell D Vorinostat in patients with advanced malignant pleural mesothelioma who have progressed on previous chemotherapy (VANTAGE-014): a phase 3, double-blind, randomised, placebo-controlled trial. *Lancet Oncol.* (2015) 16:447–56. 10.1016/S1470-2045(15)70056-2 25800891

[28] LaFave LM Beguelin W Koche R Teater M Spitzer B Chramiec A Loss of BAP1 function leads to EZH2-dependent transformation. *Nat Med.* (2015) 21:1344–9. 10.1038/nm.3947 26437366 PMC4636469

[29] Marchesi I Bagella L. Targeting Enhancer of Zeste Homolog 2 as a promising strategy for cancer treatment. *World J Clin Oncol.* (2016) 7:135–48. 10.5306/wjco.v7.i2.135 27081636 PMC4826959

[30] Zauderer MG Szlosarek PW Moulec SL Popat S Taylor P Planchard D Safety and efficacy of tazemetostat, an enhancer of zeste-homolog 2 inhibitor, in patients with relapsed or refractory malignant mesothelioma. *J Clin Oncol.* (2020) 38:9058. 10.1200/JCO.2020.38.15_suppl.9058 32753890

[31] Schoumacher M Le Corre S Houy A Mulugeta E Stern MH Roman-Roman S Uveal melanoma cells are resistant to EZH2 inhibition regardless of BAP1 status. *Nat Med.* (2016) 22:577–8. 10.1038/nm.4098 27270772

[32] Arzt L Quehenberger F Halbwedl I Mairinger T Popper HH. BAP1 protein is a progression factor in malignant pleural mesothelioma. *Pathol Oncol Res.* (2014) 20:145–51. 10.1007/s12253-013-9677-2 23963927

[33] Baumann F Flores E Napolitano A Kanodia S Taioli E Pass H Mesothelioma patients with germline BAP1 mutations have 7-fold improved long-term survival. *Carcinogenesis.* (2015) 36:76–81. 10.1093/carcin/bgu227 25380601 PMC4291047

[34] Broz ML Binnewies M Boldajipour B Nelson AE Pollack JL Erle DJ Dissecting the tumor myeloid compartment reveals rare activating antigen-presenting cells critical for T cell immunity. *Cancer Cell.* (2014) 26:638–52. 10.1016/j.ccell.2014.09.007 25446897 PMC4254577

[35] Li W Cooper J Karajannis MA Giancotti FG. Merlin: a tumour suppressor with functions at the cell cortex and in the nucleus. *EMBO Rep.* (2012) 13:204–15. 10.1038/sj.embor.2012.11 22482125 PMC3323126

[36] Yu F-X Zhao B Guan K-L. Hippo pathway in organ size control, tissue homeostasis, and cancer. *Cell.* (2015) 163:811–28. 10.1016/j.cell.2015.10.044 26544935 PMC4638384

[37] Li W Cooper J Zhou L Yang C Erdjument-Bromage H Zagzag D Merlin/NF2 loss-driven tumorigenesis linked to CRL4(DCAF1)-mediated inhibition of the hippo pathway kinases Lats1 and 2 in the nucleus. *Cancer Cell.* (2014) 26:48–60. 10.1016/j.ccr.2014.05.001 25026211 PMC4126592

[38] Harvey KF Zhang X Thomas DM. The Hippo pathway and human cancer. *Nat Rev Cancer.* (2013) 13:246–57. 10.1038/nrc3458 23467301

[39] Tranchant R Quetel L Tallet A Meiller C Renier A de Koning L Co-occurring mutations of tumor suppressor genes, LATS2 and NF2, in malignant pleural mesothelioma. *Clin Cancer Res.* (2017) 23:3191–202. 10.1158/1078-0432.CCR-16-1971 28003305

[40] Benhamouche S Curto M Saotome I Gladden AB Liu CH Giovannini M Nf2/Merlin controls progenitor homeostasis and tumorigenesis in the liver. *Genes Dev.* (2010) 24:1718–30. 10.1101/gad.1938710 20675406 PMC2922501

[41] Zhang WQ Dai YY Hsu PC Wang H Cheng L Yang YL Targeting YAP in malignant pleural mesothelioma. *J Cell Mol Med.* (2017) 21:2663–76. 10.1111/jcmm.13182 28470935 PMC5661117

[42] Dasari VR Mazack V Feng W Nash J Carey DJ Gogoi R. Verteporfin exhibits YAP-independent anti-proliferative and cytotoxic effects in endometrial cancer cells. *Oncotarget.* (2017) 8:28628–40. 10.18632/oncotarget.15614 28404908 PMC5438678

[43] Tranchant R Quetel L Montagne F De Wolf J Meiller C De Koning L Assessment of signaling pathway inhibitors and identification of predictive biomarkers in malignant pleural mesothelioma. *Lung Cancer.* (2018) 126:15–24. 10.1016/j.lungcan.2018.10.015 30527180

[44] White SM Avantaggiati ML Nemazanyy I Di Poto C Yang Y Pende M YAP/TAZ inhibition induces metabolic and signaling rewiring resulting in targetable vulnerabilities in NF2-deficient tumor cells. *Dev Cell.* (2019) 49:425–43e9. 10.1016/j.devcel.2019.04.014 31063758 PMC6524954

[45] Poulikakos PI Xiao GH Gallagher R Jablonski S Jhanwar SC Testa JR. Re-expression of the tumor suppressor NF2/merlin inhibits invasiveness in mesothelioma cells and negatively regulates FAK. *Oncogene.* (2006) 25:5960–8. 10.1038/sj.onc.1209587 16652148

[46] Seguin L Desgrosellier JS Weis SM Cheresh DA. Integrins and cancer: regulators of cancer stemness, metastasis, and drug resistance. *Trends Cell Biol.* (2015) 25:234–40. 10.1016/j.tcb.2014.12.006 25572304 PMC4380531

[47] Shapiro IM Kolev VN Vidal CM Kadariya Y Ring JE Wright Q Merlin deficiency predicts FAK inhibitor sensitivity: a synthetic lethal relationship. *Sci Transl Med.* (2014) 6:237ra68. 10.1126/scitranslmed.3008639 24848258 PMC4165339

[48] Kato T Sato T Yokoi K Sekido Y. E-cadherin expression is correlated with focal adhesion kinase inhibitor resistance in Merlin-negative malignant mesothelioma cells. *Oncogene.* (2017) 36:5522–31. 10.1038/onc.2017.147 28553954

[49] Soria JC Gan HK Blagden SP Plummer R Arkenau HT Ranson M A phase I, pharmacokinetic and pharmacodynamic study of GSK2256098, a focal adhesion kinase inhibitor, in patients with advanced solid tumors. *Ann Oncol.* (2016) 27:2268–74. 10.1093/annonc/mdw427 27733373

[50] Fennell DA Baas P Taylor P Nowak AK Gilligan D Nakano T Maintenance defactinib versus placebo after first-line chemotherapy in patients with merlin-stratified pleural mesothelioma: COMMAND-A double-blind, randomized, phase II study. *J Clin Oncol.* (2019) 37:790–8. 10.1200/JCO.2018.79.0543 30785827

[51] Quetel L Meiller C Assie JB Blum Y Imbeaud S Montagne F Genetic alterations of malignant pleural mesothelioma: association with tumor heterogeneity and overall survival. *Mol Oncol.* (2020) 14:1207–23. 10.1002/1878-0261.12651 32083805 PMC7266286

[52] Krug LM Wozniak AJ Kindler HL Feld R Koczywas M Morero JL Randomized phase II trial of pemetrexed/cisplatin with or without CBP501 in patients with advanced malignant pleural mesothelioma. *Lung Cancer.* (2014) 85:429–34. 10.1016/j.lungcan.2014.06.008 25047675

[53] Xu D Liang SQ Yang H Bruggmann R Berezowska S Yang Z CRISPR screening identifies WEE1 as a combination target for standard chemotherapy in malignant pleural mesothelioma. *Mol Cancer Ther.* (2020) 19:661–72. 10.1158/1535-7163.MCT-19-0724 31694888

[54] Joseph NM Chen YY Nasr A Yeh I Talevich E Onodera C Genomic profiling of malignant peritoneal mesothelioma reveals recurrent alterations in epigenetic regulatory genes BAP1, SETD2, and DDX3X. *Mod Pathol.* (2017) 30:246–54. 10.1038/modpathol.2016.188 27813512 PMC5288276

[55] Ding H Zhao J Zhang Y Yu J Liu M Li X Systematic analysis of drug vulnerabilities conferred by tumor suppressor loss. *Cell Rep.* (2019) 27:3331–44e6. 10.1016/j.celrep.2019.05.043 31189115

[56] Tallet A Nault JC Renier A Hysi I Galateau-Salle F Cazes A Overexpression and promoter mutation of the TERT gene in malignant pleural mesothelioma. *Oncogene.* (2014) 33:3748–52. 10.1038/onc.2013.351 23975423

[57] Pirker C Bilecz A Grusch M Mohr T Heidenreich B Laszlo V Telomerase reverse transcriptase promoter mutations identify a genomically defined and highly aggressive human pleural mesothelioma subgroup. *Clin Cancer Res.* (2020) 26:3819–30. 10.1158/1078-0432.CCR-19-3573 32317288

[58] Solimini NL Luo J Elledge SJ. Non-oncogene addiction and the stress phenotype of cancer cells. *Cell.* (2007) 130:986–8. 10.1016/j.cell.2007.09.007 17889643

[59] Luo J Solimini NL Elledge SJ. Principles of cancer therapy: oncogene and non-oncogene addiction. *Cell.* (2009) 136:823–37. 10.1016/j.cell.2009.02.024 19269363 PMC2894612

[60] Mezzapelle R Miglio U Rena O Paganotti A Allegrini S Antona J Mutation analysis of the EGFR gene and downstream signalling pathway in histologic samples of malignant pleural mesothelioma. *Br J Cancer.* (2013) 108:1743–9. 10.1038/bjc.2013.130 23558893 PMC3668472

[61] Garland LL Rankin C Gandara DR Rivkin SE Scott KM Nagle RB Phase II study of erlotinib in patients with malignant pleural mesothelioma: a Southwest oncology group study. *J Clin Oncol.* (2007) 25:2406–13. 10.1200/JCO.2006.09.7634 17557954

[62] Mak G Soria JC Blagden SP Plummer R Fleming RA Nebot N A phase Ib dose-finding, pharmacokinetic study of the focal adhesion kinase inhibitor GSK2256098 and trametinib in patients with advanced solid tumours. *Br J Cancer.* (2019) 120:975–81. 10.1038/s41416-019-0452-3 30992546 PMC6735221

[63] Zhou S Liu L Li H Eilers G Kuang Y Shi S Multipoint targeting of the PI3K/mTOR pathway in mesothelioma. *Br J Cancer.* (2014) 110:2479–88. 10.1038/bjc.2014.220 24762959 PMC4021537

[64] Hartman ML Esposito JM Yeap BY Sugarbaker DJ. Combined treatment with cisplatin and sirolimus to enhance cell death in human mesothelioma. *J Thorac Cardiovasc Surg.* (2010) 139:1233–40. 10.1016/j.jtcvs.2009.06.027 19853261

[65] Hoda MA Mohamed A Ghanim B Filipits M Hegedus B Tamura M Temsirolimus inhibits malignant pleural mesothelioma growth in vitro and in vivo: synergism with chemotherapy. *J Thorac Oncol.* (2011) 6:852–63. 10.1097/JTO.0b013e31820e1a25 21358348

[66] Ou SH Moon J Garland LL Mack PC Testa JR Tsao AS SWOG S0722: phase II study of mTOR inhibitor everolimus (RAD001) in advanced malignant pleural mesothelioma (MPM). *J Thorac Oncol.* (2015) 10:387–91. 10.1097/JTO.0000000000000360 25611229 PMC4304604

[67] Cipriani NA Abidoye OO Vokes E Salgia R. MET as a target for treatment of chest tumors. *Lung Cancer.* (2009) 63:169–79. 10.1016/j.lungcan.2008.06.011 18672314 PMC2659375

[68] Ou WB Corson JM Flynn DL Lu WP Wise SC Bueno R AXL regulates mesothelioma proliferation and invasiveness. *Oncogene.* (2011) 30:1643–52. 10.1038/onc.2010.555 21132014

[69] Marek LA Hinz TK von Massenhausen A Olszewski KA Kleczko EK Boehm D Nonamplified FGFR1 is a growth driver in malignant pleural mesothelioma. *Mol Cancer Res.* (2014) 12:1460–9. 10.1158/1541-7786.MCR-14-0038 24966347 PMC4201974

[70] Jacobson BA De A Kratzke MG Patel MR Jay-Dixon J Whitson BA Activated 4E-BP1 represses tumourigenesis and IGF-I-mediated activation of the eIF4F complex in mesothelioma. *Br J Cancer.* (2009) 101:424–31. 10.1038/sj.bjc.6605184 19603014 PMC2720234

[71] Kothmaier H Quehenberger F Halbwedl I Morbini P Demirag F Zeren H EGFR and PDGFR differentially promote growth in malignant epithelioid mesothelioma of short and long term survivors. *Thorax.* (2008) 63:345–51. 10.1136/thx.2007.085241 18086752

[72] Strizzi L Catalano A Vianale G Orecchia S Casalini A Tassi G Vascular endothelial growth factor is an autocrine growth factor in human malignant mesothelioma. *J Pathol.* (2001) 193:468–75. 10.1002/path.824 11276005

[73] Bertino P Porta C Barbone D Germano S Busacca S Pinato S Preliminary data suggestive of a novel translational approach to mesothelioma treatment: imatinib mesylate with gemcitabine or pemetrexed. *Thorax.* (2007) 62:690–5. 10.1136/thx.2006.069872 17311837 PMC2117287

[74] Bertino P Piccardi F Porta C Favoni R Cilli M Mutti L Imatinib mesylate enhances therapeutic effects of gemcitabine in human malignant mesothelioma xenografts. *Clin Cancer Res.* (2008) 14:541–8. 10.1158/1078-0432.CCR-07-1388 18223230

[75] Scherpereel A Wallyn F Albelda SM Munck C. Novel therapies for malignant pleural mesothelioma. *Lancet Oncol.* (2018) 19:e161–72. 10.1016/s1470-2045(18)30100-1 29508763

[76] Buikhuisen WA Burgers JA Vincent AD Korse CM van Klaveren RJ Schramel FM Thalidomide versus active supportive care for maintenance in patients with malignant mesothelioma after first-line chemotherapy (NVALT 5): an open-label, multicentre, randomised phase 3 study. *Lancet Oncol.* (2013) 14:543–51. 10.1016/S1470-2045(13)70125-6 23583604

[77] Scagliotti GV Gaafar R Nowak AK Nakano T van Meerbeeck J Popat S Nintedanib in combination with pemetrexed and cisplatin for chemotherapy-naive patients with advanced malignant pleural mesothelioma (LUME-Meso): a double-blind, randomised, placebo-controlled phase 3 trial. *Lancet Respir Med.* (2019) 7:569–80. 10.1016/s2213-2600(19)30139-0 31103412

[78] Yadav RK Chae SW Kim HR Chae HJ. Endoplasmic reticulum stress and cancer. *J Cancer Prev.* (2014) 19:75–88. 10.15430/JCP.2014.19.2.75 25337575 PMC4204165

[79] Senft D Ronai ZA. UPR, autophagy, and mitochondria crosstalk underlies the ER stress response. *Trends Biochem Sci.* (2015) 40:141–8. 10.1016/j.tibs.2015.01.002 25656104 PMC4340752

[80] Hetz C. The unfolded protein response: controlling cell fate decisions under ER stress and beyond. *Nat Rev Mol Cell Biol.* (2012) 13:89–102. 10.1038/nrm3270 22251901

[81] Clarke HJ Chambers JE Liniker E Marciniak SJ. Endoplasmic reticulum stress in malignancy. *Cancer Cell.* (2014) 25:563–73. 10.1016/j.ccr.2014.03.015 24823636

[82] Hetz C Chevet E Oakes SA. Proteostasis control by the unfolded protein response. *Nat Cell Biol.* (2015) 17:829–38. 10.1038/ncb3184 26123108 PMC5546321

[83] Dalton LE Clarke HJ Knight J Lawson MH Wason J Lomas DA The endoplasmic reticulum stress marker CHOP predicts survival in malignant mesothelioma. *Br J Cancer.* (2013) 108:1340–7. 10.1038/bjc.2013.66 23412101 PMC3619254

[84] Xu D Liang SQ Yang H Luthi U Riether C Berezowska S Increased sensitivity to apoptosis upon endoplasmic reticulum stress-induced activation of the unfolded protein response in chemotherapy-resistant malignant pleural mesothelioma. *Br J Cancer.* (2018) 119:65–75. 10.1038/s41416-018-0145-3 29921948 PMC6035279

[85] Xu D Yang H Yang Z Berezowska S Gao Y Liang SQ Endoplasmic reticulum stress signaling as a therapeutic target in malignant pleural mesothelioma. *Cancers (Basel).* (2019) 11:1502. 10.3390/cancers11101502 31597321 PMC6827154

[86] Juin P Geneste O Gautier F Depil S Campone M. Decoding and unlocking the BCL-2 dependency of cancer cells. *Nat Rev Cancer.* (2013) 13:455–65. 10.1038/nrc3538 23783119

[87] Montero J Letai A. Why do BCL-2 inhibitors work and where should we use them in the clinic? *Cell Death Differ.* (2018) 25:56–64. 10.1038/cdd.2017.183 29077093 PMC5729538

[88] Merino D Kelly GL Lessene G Wei AH Roberts AW Strasser A. BH3-mimetic drugs: blazing the trail for new cancer medicines. *Cancer Cell.* (2018) 34:879–91. 10.1016/j.ccell.2018.11.004 30537511

[89] Fennell DA Rudd RM. Defective core-apoptosis signalling in diffuse malignant pleural mesothelioma: opportunities for effective drug development. *Lancet Oncol.* (2004) 5:354–62. 10.1016/S1470-2045(04)01492-5 15172356

[90] Mohiuddin I Cao X Fang B Nishizaki M Smythe WR. Significant augmentation of pro-apoptotic gene therapy by pharmacologic Bcl-xL down-regulation in mesothelioma. *Cancer Gene Ther.* (2001) 8:547–54. 10.1038/sj.cgt.7700332 11571532

[91] Barbone D Ryan JA Kolhatkar N Chacko AD Jablons DM Sugarbaker DJ The Bcl-2 repertoire of mesothelioma spheroids underlies acquired apoptotic multicellular resistance. *Cell Death Dis.* (2011) 2:e174. 10.1038/cddis.2011.58 21697949 PMC3169000

[92] Cao X Yap JL Newell-Rogers MK Peddaboina C Jiang W Papaconstantinou HT The novel BH3 alpha-helix mimetic JY-1-106 induces apoptosis in a subset of cancer cells (lung cancer, colon cancer and mesothelioma) by disrupting Bcl-xL and Mcl-1 protein-protein interactions with Bak. *Mol Cancer.* (2013) 12:42. 10.1186/1476-4598-12-42 23680104 PMC3663763

[93] Jackson MR Ashton M Koessinger AL Dick C Verheij M Chalmers AJ. Mesothelioma cells depend on the anti-apoptotic protein Bcl-xL for survival and are sensitized to ionizing radiation by BH3-mimetics. *Int J Radiat Oncol Biol Phys.* (2019) 106:867–77. 10.1016/j.ijrobp.2019.11.029 31786278

[94] Kim MC Hwang SH Kim NY Lee HS Ji S Yang Y Hypoxia promotes acquisition of aggressive phenotypes in human malignant mesothelioma. *BMC Cancer.* (2018) 18:819. 10.1186/s12885-018-4720-z 30111297 PMC6094475

[95] Kaira K Serizawa M Koh Y Takahashi T Hanaoka H Oriuchi N Relationship between 18F-FDG uptake on positron emission tomography and molecular biology in malignant pleural mesothelioma. *Eur J Cancer.* (2012) 48:1244–54. 10.1016/j.ejca.2012.01.016 22330319

[96] Nabavi N Bennewith KL Churg A Wang Y Collins CC Mutti L. Switching off malignant mesothelioma: exploiting the hypoxic microenvironment. *Genes Cancer.* (2016) 7:340–54. 10.18632/genesandcancer.124 28191281 PMC5302036

[97] Bristow RG Hill RP. Hypoxia and metabolism. Hypoxia, DNA repair and genetic instability. *Nat Rev Cancer.* (2008) 8:180–92. 10.1038/nrc2344 18273037

[98] Tsherniak A Vazquez F Montgomery PG Weir BA Kryukov G Cowley GS Defining a cancer dependency map. *Cell.* (2017) 170:564–76e16. 10.1016/j.cell.2017.06.010 28753430 PMC5667678

[99] Shibue T Weinberg RA. EMT, CSCs, and drug resistance: the mechanistic link and clinical implications. *Nat Rev Clin Oncol.* (2017) 14:611–29. 10.1038/nrclinonc.2017.44 28397828 PMC5720366

[100] Abdullah LN Chow EK. Mechanisms of chemoresistance in cancer stem cells. *Clin Transl Med.* (2013) 2:3. 10.1186/2001-1326-2-3 23369605 PMC3565873

[101] Pasdar EA Smits M Stapelberg M Bajzikova M Stantic M Goodwin J Characterisation of mesothelioma-initiating cells and their susceptibility to anti-cancer agents. *PLoS One.* (2015) 10:e0119549. 10.1371/journal.pone.0119549 25932953 PMC4416766

[102] Wu L Blum W Zhu CQ Yun Z Pecze L Kohno M Putative cancer stem cells may be the key target to inhibit cancer cell repopulation between the intervals of chemoradiation in murine mesothelioma. *BMC Cancer.* (2018) 18:471. 10.1186/s12885-018-4354-1 29699510 PMC5921988

[103] Cortes-Dericks L Froment L Boesch R Schmid RA Karoubi G. Cisplatin-resistant cells in malignant pleural mesothelioma cell lines show ALDH(high)CD44(+) phenotype and sphere-forming capacity. *BMC Cancer.* (2014) 14:304. 10.1186/1471-2407-14-304 24884875 PMC4021184

[104] Jackson SP Bartek J. The DNA-damage response in human biology and disease. *Nature.* (2009) 461:1071–8. 10.1038/nature08467 19847258 PMC2906700

[105] Curtin NJ. DNA repair dysregulation from cancer driver to therapeutic target. *Nat Rev Cancer.* (2012) 12:801–17. 10.1038/nrc3399 23175119

[106] Lord CJ Ashworth A. The DNA damage response and cancer therapy. *Nature.* (2012) 481:287–94. 10.1038/nature10760 22258607

[107] Hassan R Morrow B Thomas A Walsh T Lee MK Gulsuner S Inherited predisposition to malignant mesothelioma and overall survival following platinum chemotherapy. *Proc Natl Acad Sci USA.* (2019) 116:9008–13. 10.1073/pnas.1821510116 30975761 PMC6500142

[108] Roe OD Anderssen E Sandeck H Christensen T Larsson E Lundgren S. Malignant pleural mesothelioma: genome-wide expression patterns reflecting general resistance mechanisms and a proposal of novel targets. *Lung Cancer.* (2010) 67:57–68. 10.1016/j.lungcan.2009.03.016 19380173

[109] Romagnoli S Fasoli E Vaira V Falleni M Pellegrini C Catania A Identification of potential therapeutic targets in malignant mesothelioma using cell-cycle gene expression analysis. *Am J Pathol.* (2009) 174:762–70. 10.2353/ajpath.2009.080721 19218339 PMC2665738

[110] Indovina P Marcelli E Di Marzo D Casini N Forte IM Giorgi F Abrogating G(2)/M checkpoint through WEE1 inhibition in combination with chemotherapy as a promising therapeutic approach for mesothelioma. *Cancer Biol Ther.* (2014) 15:380–8. 10.4161/cbt.27623 24365782 PMC3979815

[111] Yang Z Klionsky DJ. Eaten alive: a history of macroautophagy. *Nat Cell Biol.* (2010) 12:814–22. 10.1038/ncb0910-814 20811353 PMC3616322

[112] Liu B Wen X Cheng Y. Survival or death: disequilibrating the oncogenic and tumor suppressive autophagy in cancer. *Cell Death Dis.* (2013) 4:e892. 10.1038/cddis.2013.422 24176850 PMC3920945

[113] Echeverry N Ziltener G Barbone D Weder W Stahel RA Broaddus VC Inhibition of autophagy sensitizes malignant pleural mesothelioma cells to dual PI3K/mTOR inhibitors. *Cell Death Dis.* (2015) 6:e1757. 10.1038/cddis.2015.124 25950487 PMC4669703

[114] Sui X Chen R Wang Z Huang Z Kong N Zhang M Autophagy and chemotherapy resistance: a promising therapeutic target for cancer treatment. *Cell Death Dis.* (2013) 4:e838. 10.1038/cddis.2013.350 24113172 PMC3824660

[115] Levy JM Thompson JC Griesinger AM Amani V Donson AM Birks DK Autophagy inhibition improves chemosensitivity in BRAF(V600E) brain tumors. *Cancer Discov.* (2014) 4:773–80. 10.1158/2159-8290.CD-14-0049 24823863 PMC4090283

[116] Follo C Cheng Y Richards WG Bueno R Broaddus VC. Inhibition of autophagy initiation potentiates chemosensitivity in mesothelioma. *Mol Carcinog.* (2018) 57:319–32. 10.1002/mc.22757 29073722

[117] Whittaker SR Theurillat JP Van Allen E Wagle N Hsiao J Cowley GS A genome-scale RNA interference screen implicates NF1 loss in resistance to RAF inhibition. *Cancer Discov.* (2013) 3:350–62. 10.1158/2159-8290.CD-12-0470 23288408 PMC3606893

[118] Linton A Cheng YY Griggs K Kirschner MB Gattani S Srikaran S An RNAi-based screen reveals PLK1, CDK1 and NDC80 as potential therapeutic targets in malignant pleural mesothelioma. *Br J Cancer.* (2014) 110:510–9. 10.1038/bjc.2013.731 24327015 PMC3899767

[119] Okonska A Buhler S Rao V Ronner M Blijlevens M van der Meulen-Muileman IH Functional genomic screen in mesothelioma reveals that loss of function of BRCA1-associated protein 1 induces chemoresistance to ribonucleotide reductase inhibition. *Mol Cancer Ther.* (2020) 19:552–63. 10.1158/1535-7163.MCT-19-0356 31619462

[120] Li L Ng SR Colon CI Drapkin BJ Hsu PP Li Z Identification of DHODH as a therapeutic target in small cell lung cancer. *Sci Transl Med.* (2019) 11:eaaw7852. 10.1126/scitranslmed.aaw7852 31694929 PMC7401885

[121] Wang C Vegna S Jin H Benedict B Lieftink C Ramirez C Inducing and exploiting vulnerabilities for the treatment of liver cancer. *Nature.* (2019) 574:268–72. 10.1038/s41586-019-1607-3 31578521 PMC6858884

[122] Hart T Chandrashekhar M Aregger M Steinhart Z Brown KR MacLeod G High-resolution CRISPR screens reveal fitness genes and genotype-specific cancer liabilities. *Cell.* (2015) 163:1515–26. 10.1016/j.cell.2015.11.015 26627737

[123] Wang T Yu H Hughes NW Liu B Kendirli A Klein K Gene essentiality profiling reveals gene networks and synthetic lethal interactions with oncogenic Ras. *Cell.* (2017) 168:890–903e15. 10.1016/j.cell.2017.01.013 28162770 PMC5445660

[124] Ruiz S Mayor-Ruiz C Lafarga V Murga M Vega-Sendino M Ortega S A genome-wide CRISPR screen identifies CDC25A as a determinant of sensitivity to ATR inhibitors. *Mol Cell.* (2016) 62:307–13. 10.1016/j.molcel.2016.03.006 27067599 PMC5029544

[125] Manguso RT Pope HW Zimmer MD Brown FD Yates KB Miller BC In vivo CRISPR screening identifies Ptpn2 as a cancer immunotherapy target. *Nature.* (2017) 547:413–8. 10.1038/nature23270 28723893 PMC5924693

[126] Blum Y Meiller C Quetel L Elarouci N Ayadi M Tashtanbaeva D Dissecting heterogeneity in malignant pleural mesothelioma through histo-molecular gradients for clinical applications. *Nat Commun.* (2019) 10:1333. 10.1038/s41467-019-09307-6 30902996 PMC6430832

[127] Oehl K Vrugt B Opitz I Meerang M. Heterogeneity in malignant pleural mesothelioma. *Int J Mol Sci.* (2018) 19:1603. 10.3390/ijms19061603 29848954 PMC6032160

[128] Boj SF Hwang CI Baker LA Chio II Engle DD Corbo V Organoid models of human and mouse ductal pancreatic cancer. *Cell.* (2015) 160:324–38. 10.1016/j.cell.2014.12.021 25557080 PMC4334572

[129] Italiano A Soria JC Toulmonde M Michot JM Lucchesi C Varga A Tazemetostat, an EZH2 inhibitor, in relapsed or refractory B-cell non-Hodgkin lymphoma and advanced solid tumours: a first-in-human, open-label, phase 1 study. *Lancet Oncol.* (2018) 19:649–59. 29650362 10.1016/S1470-2045(18)30145-1

[130] Walter RFH Sydow SR Berg E Kollmeier J Christoph DC Christoph S Bortezomib sensitivity is tissue dependent and high expression of the 20S proteasome precludes good response in malignant pleural mesothelioma. *Cancer Manag Res.* (2019) 11:8711–20. 10.2147/CMAR.S194337 31576173 PMC6765394

[131] Fennell DA Danson S Woll PJ Forster M Talbot D Child J Ganetespib in combination with pemetrexed-platinum chemotherapy in patients with pleural mesothelioma (MESO-02): a phase Ib trial. *Clin Cancer Res.* (2020): 10.1158/1078-0432.CCR-20-1306 [Epub ahead of print]. 32669375

